# A dataset of food choice motives among adults consumers in Brazil: The use of Food Choice Questionnaire

**DOI:** 10.1016/j.dib.2021.107703

**Published:** 2021-12-12

**Authors:** Camila de Mello Marsola, Luís Miguel Cunha, Joana Pereira Carvalho-Ferreira, Diogo Thimoteo da Cunha

**Affiliations:** a Laboratório Multidisciplinar em Alimentos e Saúde. Faculdade de Ciências Aplicadas, Universidade Estadual de Campinas—UNICAMP, R. Pedro Zaccaria, 1300, Limeira, SP 13484-350, Brazil; b GreenUPorto, DGAOT, Faculty of Sciences, University of Porto, Campus Agrário de Vairão, R. da Agrária, 747, Vila do Conde 4485–646, Portugal

**Keywords:** Food choice, Food consumption, FCQ, Food behavior

## Abstract

The Food Choice Questionnaire (FCQ) was applied to assess the motivations for daily food choices and associated factors in a Brazilian sample. Data were collected from January to July 2019 from 525 individuals over 18 years old recruited face-to-face in different places (e.g., university, public squares, health posts), using a convenient, intentional, and reasoned sampling. In addition to the FCQ, socioeconomic data were collected from printed questionnaires. Answers were given using a seven-point scale, ranging from (1) strongly disagree to (7) strongly agree. After Confirmatory factor analysis led to the rejection of the original FCQ structure, exploratory factor analysis was performed. Eight factors were extracted and named: nutritional composition, mood, health, sensory appeal, price, preparation convenience, familiarity, and purchase convenience. Other analyses were performed and led to a previously published discussion about food choice criteria hierarchy and associated factors. Researchers and practitioners can further use data from this survey in science and practice. These data can be useful for product development, nutritional counseling, and public health policies development. Furthermore, the FCQ is a widely used instrument, and comparisons between results obtained in different samples can bring meaningful contributions to the study of consumer behavior.

## Specifications Table

**Table:** 

Subject	Food Science
Specific subject area	Behavioral drivers of food choice; Consumer's science
Type of data	Table
How the data were acquired	Data from a cross-section study made in Brazil [1]. The participants were face-to-face recruited in different locations (e.g., health posts, public squares, university) to increase sample heterogeneity. The sample was planned with quotas for age and sex to be equivalent to population characteristics. The sample was recruited personally and aimed to include the following characteristics in certain proportions: 50% female representatives, 90% adult and 10% elderly, and 50% who had an income of less than four minimum wages in Brazil. The participants were all recruited in the Limeira region (São Paulo – Brazil). The Food Choice Questionnaire (FCQ) is a validated questionnaire with 36 indicators. The FCQ assesses several factors that influence food choice: ``health'', ``sensory appeal'', ``price'', ``convenience'', ``mood'', ``natural content'', ``weight control'', ``familiarity'' and ``ethical concern''. Answers are given using a 7-point Likert scale, ranging from 1 - strongly disagree to 7-strongly agree. The FCQ was developed initially by Steptoe et al. [2], and it was adapted and validated for Brazilian culture by Heitor et al. [3]. A pen-and-paper structured questionnaire was applied.
Data format	Raw and cleaned
Description of data collection	The recruitment was carried out in different locations in the Limeira region (São Paulo State – Brazil) to reach different socioeconomic contexts. The data were collected from February to July of 2019. The food choice questionnaire was applied in printed version, and the participants answered using paper and pencil. The data collection was conducted by trained researchers with a higher degree in Nutrition or Nutrition students. All respondents answered the questionnaire alone, without interferences from the researcher or any other person. A field supervisor checked the data collection and questionnaires.
Data source location	Country: Brazil City/State: Limeira region, São Paulo
Data accessibility	Repository name: Mendeley Data Data identification number: 10.17632/4g5t95nnxf.1 Direct URL to data: 10.17632/4g5t95nnxf.1
Related research article	Marsola, C.M.; Cunha, L.M.; Carvalho-Ferreira, J.P.; da Cunha, D.T. Factors Underlying Food Choice Motives in a Brazilian Sample: The Association with Socioeconomic Factors and Risk Perceptions about Chronic Diseases. Foods 2020, 9, 1114. doi: 10.3390/foods9081114

## Value of the Data

• These data present information on socioeconomic characteristics and food choice motives using the Food Choice Questionnaire (FCQ). Researchers and practitioners could use these data for both science and practice. Data from food choice motives are helpful for designing new products, shaping food marketing, understanding food behaviors, and developing targeted nutrition counseling.
• These data provides valuable information to government organizations and non-government organizations to formulate policies and strategies to improve food security and nutritional status.
• Food Choice Questionnaire (FCQ) is a widely used questionnaire to assess food choice motives [4]. With this dataset, researchers from different countries or different Brazil regions could compare their data with these data. Modifications in the FCQ structure are typical in diverse populations. Cultural diversity explains such differences, including regionalism, food accessibility, food culture, and many social characteristics [5,6]. With this data, it is possible to verify different factor structures, conduct multiple-group analysis and discuss cultural differences of food choice.
• These data was also used as an explanatory variable in another study [7]. The FCQ can be used to identify food choice motives and also to explain other food-related behaviors.
• These data was collected before the COVID-19 pandemic. It is known that the pandemic changed the food environment and practices [8,9]. This FCQ data can be compared with actual data to verify the real impact of the pandemic on food choices.

## 1 Data Description

**Fig. 1:**
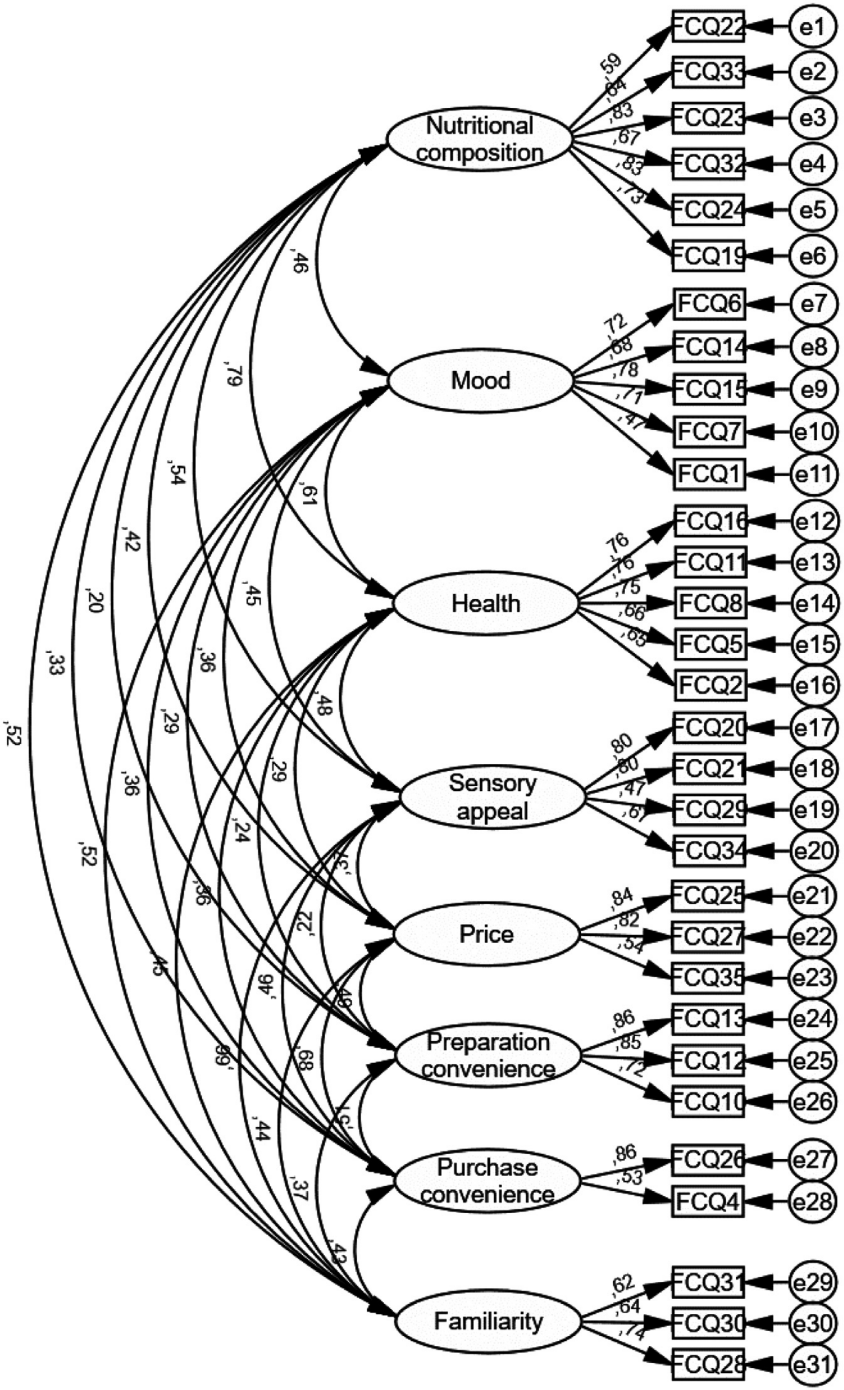
Confirmatory factor analysis of the final model.

The dataset included 525 respondents over 18 years old from the Limeira region. The data compromise responses from both sexes. Socioeconomic information such as marital status, children, education level, and family income are also included. The first data file contains the raw data of socioeconomic status and Food Choice Questionnaire responses. The score of each indicator is provided. The second data file consists of a clean database by removing the missing values on any FCQ indicators. This second database provides the mean values of FCQ extracted factors after exploratory (EFA) and confirmatory factor analysis (CFA). Eight factors were extracted: nutritional composition, mood, health, sensory appeal, price, preparation convenience, familiarity, and purchase convenience. Fig. 1 presents the CFA of the final model.

A third file with the codebook was included, describing all the codes from nominal and categorical data. Finally, the English version of FCQ was available.

## 2 Experimental Design, Materials and Methods

It was a cross-sectional study in Brazil. The data was collected for six months, from February to July of 2019.

### 2.1 Sample

A pilot study with fifty participants was conducted at first to assist in sample estimation. From the results observed in this pilot study, the sample was calculated considering: the probability of failing to reject a false null hypothesis (β-error) = 1%; The probability of falsely rejecting a true null hypothesis (α-error) = 5%; effect size (d) = 0.30, which led us to a sample of 204 participants. Aiming to improve the data fit, a more robust sample of 525 participants was chosen. Participants over 18 years of age were recruited. The sample needed was defined based on Kyriazos [10] recommendations, including studies with communalities close to 0.50, factors with multiple indicators (>3), factors with expected high loadings (>0.70), Standard root mean squared residual (SRMR) < 0.10. The sample showed 99% of statistical power when considering alpha error = 0.05 and RMSEA <0.07 [11].

The recruitment was conducted in diverse locations to reach different socioeconomic contexts, including universities, health posts, public squares, and condominiums. The sample for this study was selected by convenient sampling, intentional and aiming to achieve certain representativeness (e.g., sex and age) [12]. The selection of the sample took place intending to reach the prevalence of specific characteristics in the population, like sex (∼50% female), age (∼89.5% adults and ∼10.5% elderly), and income (∼50% with lower income – income below four times the minimum wage). The researchers identified specific locations to sample specific population niches (e.g., low-income consumers). Regarding education, it was categorized into two categories: low, in which individuals with incomplete primary education to complete secondary education were included, and high, in which individuals with complete secondary education to complete higher education were included. Concerning family income, it was also categorized into two categories: low, being considered those with income from one to four monthly minimum wages, and high, considered here those with income above five monthly minimum wages.

The interviews were conducted by three trained researchers, who applied the questionnaires face-to-face. Participants were approached personally. First, they were asked if they were willing to participate in a survey. Afterward, the research was briefly explained, and a consent form was presented.

### 2.2 Data collection

A pen-and-paper structured questionnaire was applied. The volunteer was oriented about the study and how to answer the questionnaire. First, to characterize the sample, data on socioeconomic status were collected, such as family income (five ratings from 1 – one to two minimum wages to 5 - five or more minimum wages), educational level (six ratings from 1 - incomplete elementary school to 6 - complete higher education), marital status (single, married, divorced and widowed), and children (yes or no). For this, we used closed questions with ordinal scales of response.

**Fig. 2:**
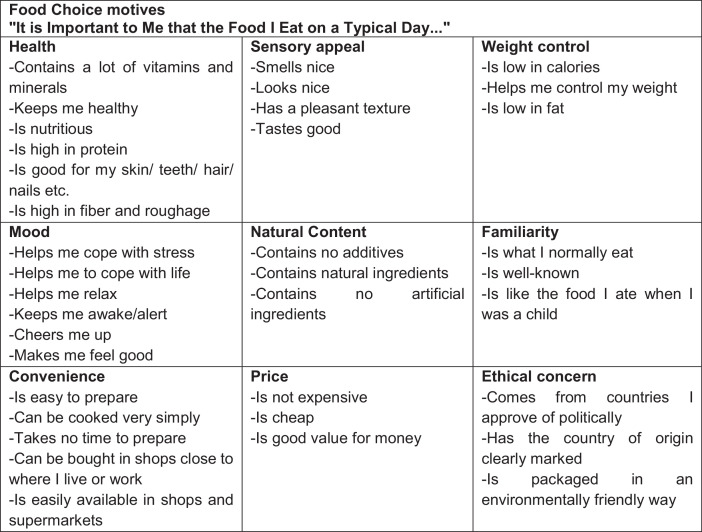
Food Choice Questionnaire original structure and indicators.

The FCQ is a multidimensional instrument developed by Steptoe et al. [2] to assess the food choice motives. The questionnaire has 36 indicators encompassing nine categories according to the proximity of food: ``health'', ``sensory appeal'', ``price'', ``convenience'', ``mood'', ``natural content'', ``weight control'', ``familiarity'' and ``ethical concern'' (Fig. 2). It was used the translated and validated version for Portuguese [3]. Answers were given using a seven-point scale, with the description in all scale points, ranging from (1) strongly disagree, (2) disagree, (3) somewhat disagree, (4) neither agree nor disagree, (5) somewhat agree, (6) agree, (7) strongly agree. All respondents answered the questionnaire alone, without interferences from the researcher or any other person. In case of doubt, the investigators aided the respondents without inducing any answer. Questionnaires were administered in a silent place. All participants were seated while completing the questionnaires.

### 2.3 Data analysis

Initially, the data were tested for normality. The Lilliefors-corrected Kolmogorov–Smirnov's test was used to verify the parametric distribution. The histogram, kurtosis, and skewness of the variables were also studied.

A CFA procedure was done using structural equation modeling (SEM) based on covariances. It was used the software AMOS (Analysis of Moment Structures) Graphics 26. This step is necessary to check if the sampled data adjust the original FCQ structure [2], as previously discussed by Cunha et al. [4]. The model adjustment was analyzed using the comparative fit index (CFI), Root-Mean-Square Error of Approximation (RMSEA), Standardized Root Mean Square Error (SRMR), and PClose, based on Hu and Bentler cutoffs [13]. The CFI calculates the relative fit of the observed model by comparing it with a base model, whose values above 0.95 indicate optimal fit and those above 0.90 indicate a good fit. The RMSEA is also a discrepancy measure, with results lower than 0.05 being expected but acceptable up to 0.08. The SRMR reports the standardized mean of the residuals and indexes less than 0.10 indicate a good fit. We also used PClose value, *p-value* of the null hypothesis that the RMSEA estimate is below 0.05 [14]. The original model presented poor fit, with CFI = 0.83, SRMR = 0.07, RMSEA = 0.07 and PClose < 0.001.

Since the sampled data did not adequately fit the FCQ original structure, an EFA was performed. The EFA was conducted in the software Statistical Package for Social Sciences – SPSS 20.0. Only indicators with a factor loading higher than 0.40 were considered. The varimax rotation was used to clarify the relationship among factors. The Kaiser–Meyer–Olkin (KMO > 0.70) was used for sample adequacy. Bartlett's test for homogeneity of variances was used to check if the variables were orthogonal. A seven-factor solution (eigenvalue > 1) were extracted, explaining 51.9% of total variance. The new FCQ factors' reliability was assessed by using Cronbach's alpha (α > 0.60) and composite reliability (> 0.70). After observing adequate reliability, the new model was submitted to another CFA, using the newly achieved structure (Fig. 1). The new FCQ structure presented a better fit than the original model in the second CFA, with CFI = 0.88, SRMR = 0.067, RMSEA = 0.06 and Pclose = 0.01. Based on this new solution, some factors were renamed based on its indicator, as depicted in Fig. 1. The original ethical concern factor was not extracted, so its indicators were excluded to improve CFA fit.

### 2.4 Steps to reproduce the results

(1) Check the validated version of the FCQ for the desired language (for more information, please check [4]);
(2) Print the questionnaires and train your research team;
(3) Recruit volunteers following your sampling plan. Apply the questionnaires personally, helping the volunteers and in a quiet place;
(4) Tabulate the data;
(5) Perform the CFA using the original structure of FCQ (Fig. 2). Check the structure fit;
(6) Perform the EFA if the CFA does not present an adequate fit;
(7) Perform a new CFA with the new structure to check the structure fit.

## Ethics Statement

All the procedures were done according to the declaration of Helsinki. All participants gave written informed consent. The University of Campinas Ethics Committee approved the protocol (CAAE number: 91222418.5.0000.5404).

## CRediT authorship contribution statement

**Camila de Mello Marsola:** Conceptualization, Formal analysis, Methodology, Investigation, Writing – original draft. **Luís Miguel Cunha:** Funding acquisition, Visualization, Writing – review & editing. **Joana Pereira Carvalho-Ferreira:** Funding acquisition, Visualization, Writing – review & editing. **Diogo Thimoteo da Cunha:** Funding acquisition, Conceptualization, Formal analysis, Project administration, Supervision, Methodology, Resources, Visualization, Writing – review & editing.

## Declaration of Competing Interest

The authors declare that they have no known competing financial interests or personal relationships that could have appeared to influence the work reported in this paper.
